# The predictive power of the National Early Warning Score (NEWS) 2, as compared to NEWS, among patients assessed by a Rapid response team: A prospective multi-centre trial

**DOI:** 10.1016/j.resplu.2021.100191

**Published:** 2021-12-24

**Authors:** Anna Thorén, Eva Joelsson-Alm, Martin Spångfors, Araz Rawshani, Thomas Kahan, Johan Engdahl, Martin Jonsson, Therese Djärv

**Affiliations:** a Department of Medicine Solna, Centre for Resuscitation Science, Karolinska Institutet, SE-171 77 Stockholm, Sweden; b Department of Clinical Physiology, Danderyd University Hospital, SE-182 88 Stockholm, Sweden; c Department of Clinical Science and Education, Karolinska Institutet, SE-118 83 Stockholm, Sweden; d Department of Anaesthesia and Intensive Care, Södersjukhuset, SE-118 83 Stockholm, Sweden; e Department of Clinical Sciences, Lund University, SE-221 84 Lund, Sweden; f Department of Anaesthesia and Intensive Care, Kristianstad Hospital, SE-291 89 Kristianstad, Sweden; g Department of Molecular and Clinical Medicine, Institute of Medicine, University of Gothenburg, SE-405 30 Gothenburg, Sweden; h Division of Cardiovascular Medicine, Department of Clinical Sciences, Danderyd University Hospital, Karolinska Institutet, SE-182 88 Stockholm, Sweden; i Department of Clinical Science and Education, Centre for Resuscitation Science, Karolinska Institutet, Södersjukhuset, SE-118 83 Stockholm, Sweden; j Department of Emergency Medicine, Karolinska University Hospital, SE- 171 64 Stockholm, Sweden

**Keywords:** National early warning score, National early warning score 2, Vital signs, Rapid response team, Mortality, In-hospital cardiac arrest, IHCA, NEWS, National Early Warning Score, RRT, Rapid response team, IHCA, In hospital cardiac arrest, AUROC, Area under the receiver operating characteristic curves, ICU, Intensive care unit, SAE, Serious adverse event, OR, Odds ratio, CI, Confidence interval

## Abstract

**Aim:**

Early identification of patients at risk of serious adverse events (SAEs) is of vital importance, yet it remains a challenging task. We investigated the predictive power of National Early Warning Score (NEWS) 2, as compared to NEWS, among patients assessed by a Rapid response team (RRT).

**Methods:**

Prospective, observational cohort study on 898 consecutive patients assessed by the RRTs in 26 Swedish hospitals. For each patient, NEWS and NEWS 2 scores were uniformly calculated by the study team. The associations of NEWS and NEWS 2 scores with unanticipated admissions to Intensive care unit (ICU), mortality and in-hospital cardiac arrests (IHCA) within 24 h, and the composite of these three events were investigated using logistic regression. The predictive power of NEWS and NEWS 2 was assessed using the area under the receiver operating characteristic (AUROC) curves.

**Results:**

The prognostic accuracy of NEWS/NEWS 2 in predicting mortality was acceptable (AUROC 0.69/0.67). In discriminating the composite outcome and unanticipated ICU admission, both NEWS and NEWS 2 were relatively weak (AUROC 0.62/0.62 and AUROC 0.59/0.60 respectively); for IHCA the performance was poor. There were no differences between NEWS and NEWS 2 as to the predictive power.

**Conclusion:**

The prognostic accuracy of NEWS 2 to predict mortality within 24 h was acceptable. However, the prognostic accuracy of NEWS 2 to predict IHCA was poor. NEWS and NEWS 2 performed similar in predicting the risk of SAEs but their performances were not sufficient for use as a risk stratification tool in patients assessed by a RRT.

## Introduction

Early identification of patients with deteriorating vital signs is of major importance in order to prevent further clinical deterioration and serious adverse events (SAEs), yet it remains a challenging task in healthcare settings worldwide. In-hospital cardiac arrest (IHCA) and unanticipated Intensive care unit (ICU) admission are SAEs associated with a high mortality,1., 2., 3., 4. and both are typically preceded by deviating vital signs.5., 6., 7., 8. Thus, early recognition and an adequate, timely intervention may save lives. This has prompted the development of early warning scores, which are recommended by the European Resuscitation Council (ERC) with the aim of preventing IHCA.^9^ Furthermore, Rapid response teams (RRTs) have been introduced to assess deteriorating patients, initiate interventions and, if needed, timely transfers to intensive care, with a view to improve prognosis.10., 11.

The National Early Warning Score (NEWS), launched in 2012 in the UK,^12^ has outperformed other early warning score instruments,^13^ and has been widely adopted worldwide. NEWS has undergone extensive validation in the UK and internationally, including Sweden.14., 15. An updated version, NEWS 2, was introduced in 2017 in order to improve precision and to facilitate early identification of sepsis.^16^ The main modifications were addition of a dedicated Sp0_2_ scoring scale (Scale 2) for use in patients with hypercapnic respiratory failure and the variable “new confusion” (including disorientation, delirium or any new onset alteration to mentation) to the Alert/Verbal/Pain/Unresponsive score, thus transformed into an Alert/Confusion/Verbal/Pain/Unresponsive score.^16^

There is limited knowledge as to the predictive power of NEWS and NEWS 2 to identify patients at risk in a selected cohort of patients being reviewed by the RRT. Furthermore, no prospective studies seem to have been published comparing the predictive ability of NEWS and NEWS 2 to identify patients at risk of SAEs. Thus, this study aimed to investigate the predictive power of NEWS 2 and to compare the ability of NEWS and NEWS2 to identify patients at risk of unanticipated ICU admission, IHCA, and mortality within 24 h of a RRT review.

## Methods

### Study design and setting

This prospective, observational multicenter study was conducted between October 22, 2019 and January 13, 2020 in 26 Swedish hospitals (Supplementary Table 1). Hospitals were eligible if they had a RRT system and had implemented either NEWS or NEWS 2. The RRTs consisted of a physician and a nurse specialist from the ICU in 24 of the participating hospitals, an ICU physician only in one hospital, and a physician, a nurse specialist and an assistant nurse from the ICU in one hospital. All hospitals except one performed RRT assessments 24/7. All patients were assessed by the RRT according to clinical practice. All variables required for calculation of both NEWS and NEWS 2 scores were collected by the RRT. For each patient the scores were uniformly calculated by the study team when analysing data since miscalculation is one main cause of error when using NEWS.14., 17. The decisions made after the RRT review regarding level of care and a possible revised decision of limitations of medical treatment, was also recorded. A follow-up was performed after > 24 hours, retrieving information from medical records on unanticipated ICU admission, IHCA, or in-hospital death within 24 h of the observation of vital parameters and RRT assessment.

### Participants

**Fig. 1:**
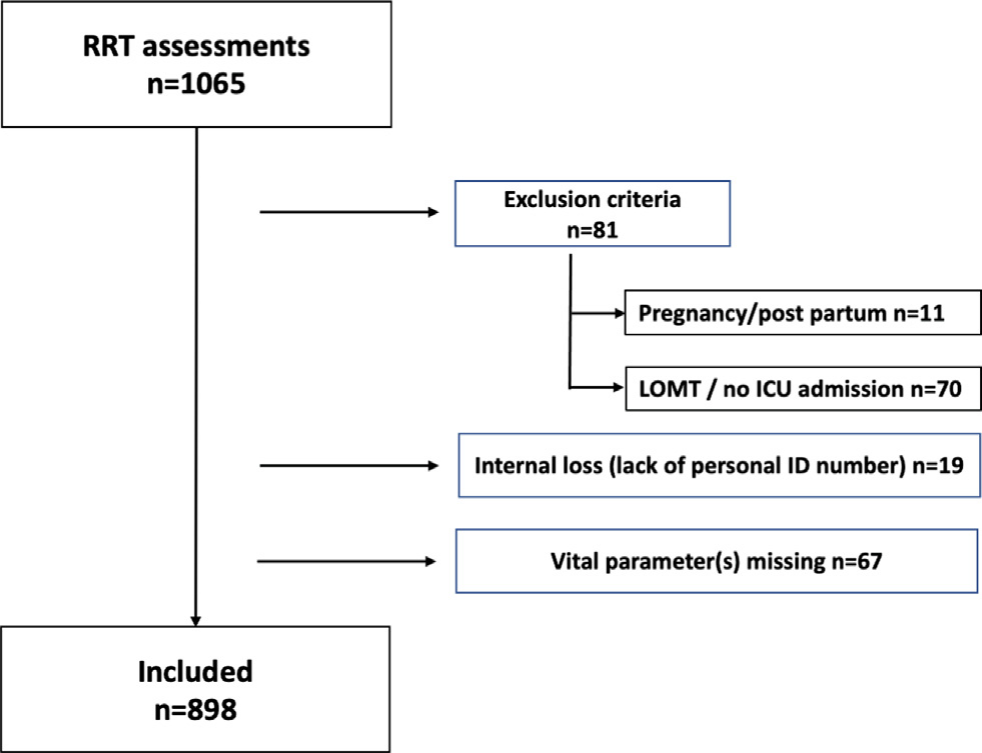
Study cohort. RRT, Rapid response team; ICU, Intensive care unit; LOMT, limitation of medical treatment.

All patients aged 18 years and older assessed by the RRTs during the inclusion period were eligible for inclusion in the study. Exclusion criteria were pregnancy and the postpartum period (i.e. 6 weeks following childbirth; n = 11), a decision of no ICU-admission prior to RRT review (n = 70) and patients being assessed as a planned follow-up after discharge from the ICU. Of a total of 1065 assessments performed during the study period, 81 met at least one exclusion criterion (Fig. 1), 19 patients were excluded due to lack of personal ID number which made it impossible to perform follow-up, and another 67 had information on at least one vital parameter missing (most frequently body temperature). Thus, 898 patients were included in the complete case analysis. Based on published results from an observational study on NEWS ^14^, we considered a study population with assessments of 1000 patients would be sufficient for the purpose of the current study.

### Definitions

The NEWS scale includes seven vital signs (respiration rate, oxygen saturation, body temperature, systolic blood pressure, heart rate and level of consciousness) scored from 0 to 3, depending on the severity of the divergence from the normal value, added into a summary score. Patients receiving supplementary oxygen, have 2 points added to their score. A score of ≥7 is a key trigger and should prompt emergency assessment by a clinical team such as the RRT.^16^ Patients are stratified into risk categories based upon the total NEWS score, which can be related to the patient́s risk of critical illness. The NEWS/NEWS 2 concept also includes a clinical response scale where different interventions are linked to the summary score.

### Outcomes

The outcomes were unanticipated ICU-admission, IHCA and in-hospital death within 24 h of an observation set of vital parameters and assessment by the RRT.13., 15., 18., 19. Since IHCA and unexpected death are infrequent events all three SAEs were analysed as a composite outcome, which is in line with the Tripod guidelines.^20^ In the event of multiple outcomes, the first was used for the analysis. The study used the definition of IHCA stated by the Swedish Registry of Cardiopulmonary Resuscitation, i.e. a hospitalized patient where cardiopulmonary resuscitation and/or defibrillation have been initiated.

### Statistical analysis

Descriptive data are presented as medians or proportions, with the appropriate dispersion measure. Logistic regression was used to predict ICU admission, IHCA and mortality as well as the combined outcome of these SAEs. The results were presented as odds ratio (OR) with 95% confidence interval (CI), adjusted for age and gender. The Hosmer & Lemeshov test was conducted to determine the goodness-of-fit. The ability of NEWS and NEWS 2 to discriminate patients at risk of IHCA and in-hospital death was evaluated by use of the area under the receiver operator characteristic curves (AUROC).^21^ Differences in AUROC between NEWS and NEWS 2 were compared by the DeLong test. The effect of the commonly used thresholds for triggering specific actions (e.g. aggregated NEWS or NEWS 2 scores ≥ 5 and ≥7, respectively) was evaluated by means of sensitivity, specificity and positive predictive values. When comparing the relative accuracy of NEWS and NEWS 2 the relative true-positive ratio (indicative of superiority in sensitivity of a test) and relative false-positive ratio (indicative of superiority in specificity of a test) were calculated.22., 23. Furthermore, the number needed to evaluate was calculated. It has been suggested that the number needed to evaluate is superior to AUROC when evaluating early warning score systems, since it better describes the balance between correct/sufficient activation and risk of alarm fatigue.22., 24. The significance level was set to a two sided probability (P < 0.05). The statistical analyses were performed with R, version 3.6.1, and with IBM SPSS Inc. version 26.0 (Chicago, IL, USA).

### Ethics

The study was approved by the Swedish Ethical Review Authority, Sweden (reference # 2019–04269). The patients included in the study is a vulnerable group, frequently suffering from critical illness, thus making it difficult for them to make a decision on participating in the study or not. However, it is of great importance to include this group of patients in the study since the outcome will improve knowledge about identification and clinical management of at-risk patients in the future. Therefore, inclusion in this non-interventional study was considered exempt from patient consent.

## Results

Descriptive data of the 898 patients included and the RRT assessment are presented in Table 1. Median age was 72 years, 42.9 % were women and the most common causes for admission to hospital were infections, followed by surgical and orthopedic conditions (Table 1 and Supplementary Table 2). Only a few patients (13.2%) had a decision on limitations of medical treatment (Table 1).

**Table 1:** Study cohort characteristics and data on RRT assessments (n = 898). Data are presented as numbers (percentages).

Age (years), median (Q1, Q3)	72 (64, 79)
Female	385 (43)
Clinical affiliation	
Medicine	359 (40)
Surgery	263 (29)
Orthopedic	83 (9.2)
Infection	76 (8.5)
Emergency Department	39 (4.3)
Geriatric	16 (1.8)
Psychiatry	6 (0.7)
Other	56 (6.2)
Diagnosis upon admission	
Surgical diseases	199 (22.7)
Infections	163 (18.1)
Orthopedic diseases	76 (8.5)
Sepsis	73 (8.1)
Dyspnoe	46 (5.1)
Malignancy	28 (3.1)
Cardiovascular diseases	25 (2.8)
Respiratory diseases	24 (2.7)
Altered level of consciousness	22 (2.4)
Catastrophic conditions	21 (2.3)
Neurological diseases	17 (1.9)
Other cause of admission	204 (23)
Primary reason for RRT call	
NEWS/NEWS 2 score	538 (60)
Concern for the patient	221 (25)
Other	139 (16)
Time of day RRT assessment	
08:00 to 17:00	673 (75)
On-call hours (17:00 to 08:00)	225 (25)
LOMT prior to RRT assessment	
Full care	780 (87)
DNACPR	111 (12)
Other	7 (0.8)

NEWS, National Early Warning Score; RRT, Rapid response team; LOMT, limitations of medical treatment; DNACPR, do not attempt cardiopulmonary resuscitation.

The majority of RRT assessments were performed during office hours, and most patients assessed by the RRT were hospitalized in wards belonging to internal medicine and surgery (Table 1). The reasons for RRT activation were the NEWS or NEWS 2 score in 59.9 %, staff concern in 24.6% and other causes in 15.5% (Table 1 and Supplementary Table 3). The median NEWS or NEWS 2 score during RRT assessment for patients remaining at ward, transferred to the High dependency unit or the ICU, respectively, are listed in Table 2. A NEWS score ≥ 7 was present among 71.2 % and a NEWS 2 score ≥ 7 was present among 72.8 % of the patients.

**Table 2:** Decisions on continued care after RRT assessment, outcomes and median scores for NEWS and NEWS 2 respectively (n = 898).

Decision on continued care after assessment, n (%)	
Immediate admission to ICU	241 (27)
Patients remaining at ward after RRT assessment	562 (63)
Patients transferred to HDU	95 (11)
Patients recieving a new LOMT	67 (7.5)
Outcomes, n (%)	
Admission to ICU within 24 h after RRT assessment	333 (37)
Cardiac arrest within 24 h of RRT assessment	10 (1.1)
Mortality within 24 h of RRT assessment	51 (5.7)
NEWS score median, (Q1, Q3)	
Immediate admission to ICU	9 (7,11)
Patients remaining at ward after RRT assessment	8 (6,10)
Patients transferred to HDU	8 (6,10)
NEWS 2 score median, (Q1, Q3)	
Immediate admission to ICU	9 (7,11)
Patients remaining at ward after RRT assessment	8 (6,10)
Patients transferred to HDU	8 (6,10)

NEWS, National Early Warning Score; RRT, Rapid response team; ICU, Intensive care unit; HDU, High dependency unit; LOMT, limitation of medical treatment.

During the study period, 26.8 % of the patients were immediately transferred to the ICU and 10.6 % to a higher level of care other than ICU (Table 2). Their NEWS and NEWS 2 scores are presented in Table 2. In some cases (7.5%), a new decision regarding limitations of medical treatment was made by the RRT.

A total of 333 patients (37.1%) were admitted to the ICU within 24 h, 10 patients (1.1%) suffered from cardiac arrest within 24 h and 51 patients (5.7%) died within 24 h of the RRT assessment (Table 2). In all, 394 patients (43.9 %) were affected by the composite outcome.

A NEWS or NEWS 2 score ≥ 7 was associated with the composite endpoint and also with mortality and ICU admission (Table 3). The OR for IHCA was not computed due to low number of cases.

**Table 3:** Logistic regression of the NEWS and NEWS 2 associations with the outcomes unanticipated ICU admission, mortality and the composite endpoint (unanticipated ICU admission, mortality or IHCA), all within 24 h of RRT assessment, using a threshold of ≥7 unadjusted/adjusted for age and gender (n = 898).

	OR	(95% CI)	P-value
ICU Admission			
NEWS ≥ 7, unadjusted	1.8	(1.3–2.5)	<0.001
NEWS ≥ 7, adjusted	1.9	(1.4–2.6)	<0.001
NEWS 2 ≥ 7, unadjusted	1.8	(1.3–2.5)	<0.001
NEWS 2 ≥ 7, adjusted	1.9	(1.4–2.6)	<0.001
Mortality			
NEWS ≥ 7, unadjusted	3.2	(1.3–7.6)	0.008
NEWS ≥ 7, adjusted	3.0	(1.3–7.2)	0.012
NEWS 2 ≥ 7, unadjusted	2.4	(1.0–5.5)	0.031
NEWS 2 ≥ 7, adjusted	2.3	(1.0–5.2)	0.047
Composite outcome			
NEWS ≥ 7, unadjusted	2.1	(1.6–2.9)	<0.001
NEWS ≥ 7, adjusted	2.2	(1.6–3.0)	<0.001
NEWS 2 ≥ 7, unadjusted	2.1	(1.5–2.9)	<0.001
NEWS 2 ≥ 7, adjusted	2.2	(1.6–3.0)	<0.001

NEWS, National Early Warning Score; ICU, Intensive care unit; RRT, Rapid response team; OR, Odds ratio; CI, Confidence interval.

The prognostic accuracy of NEWS and NEWS 2 according to low risk and medium/high risk by NEWS (according to NEWS or NEWS 2 scores ≥ 5 and ≥7 respectively) is presented in Table 4.

**Table 4:** Prognostic accuracy for NEWS and NEWS 2 for the composite outcome unanticipated ICU admission, mortality or IHCA all within 24 h of RRT assessment) (n = 898).

	NEWS ≥ 5	NEWS 2 ≥ 5	NEWS ≥ 7	NEWS 2 ≥ 7
	(n = 801, 89.2%)	(n = 784, 87.3%)	(n = 639, 71.2%)	(n = 654, 72.8%)
Sensitivity percent (95% CI)	93.2 (90.1–95.5)	91.8 (88.5–94.4)	80.0 (75.5–84.0)	81.1 (76.7–85.0)
Specificity percent (95% CI)	13.5 (10.7–16.7)	15.8(12.8–19.1)	34.9 (30.8–39.1)	32.8 (28.9–37.0)
Diagnostic accuracy percent (95% CI)	45.9(42.6–49.2)	46.7 (43.4–50.0)	53.2 (49.9–56.5)	52.4 (49.1–55.8)
Positive predictive value % (95% CI)	42.4 (39.0–46.0)	42.7 (39.2–46.3)	45.7 (41.8–49.6)	45.3(41.4–49.2)
Negative predictive value % (95% CI)	74.2 (64.3–82.6)	73.7 (64.6–81.5)	71.8 (65.9–77.2)	71.7 (65.6–77.3)
Positive likelihood ratio	1.08 (1.03–1.13)	1.09 (1.04–1.14)	1.23 (1.13–1.33)	1.21 (1.12–1.30)
Negative likelihood ratio	0.51 (0.33–0.078)	0.52 (0.35–0.77)	0.57 (0.45–0.72)	0.58 (0.45–0.74)
Percentage of subjects with outcome ruled out (95% CI)	10.8 (8.8–13.0)	12.7 (10.6–15.1)	28.8 (25.9–31.9)	27.2(24.3–30.2)
Percentage of subjects with outcome ruled in (95% CI)	89.2 (87.0–91.2)	87.3 (84.9–89.4)	71.2 (68.1–74.1)	72.8 (69.8–75.7)
Diagnostic OR (95% CI)	2.1 (1.3–3.4)	2.1 (1.3–3.2)	2.1 (1.6–2.9)	2.1 (1.5–2.9)
NNE (95% CI)	15	13	7	7
rTPR		0.98		1.01
rFPR		0.97		1.03

NEWS, National Early Warning Score; RRT, Rapid response team; ICU, Intensive care unit; IHCA, in hospital cardiac arrest; CI, Confidence interval; NNE, number needed to evaluate; rTPR, relative true positive ratio (NEWS2 TPR/NEWS TPR); rFPR, relative false positive ratio (NEWS 2 FPR/NEWS FPR); OR, Odds ratio.

There was no difference in the number needed to evaluate between NEWS and NEWS 2 using a threshold of ≥7 (number needed to evaluate 7 for both scales). Using a threshold of ≥5 the number needed to evaluate was 15 (NEWS) and 13 (NEWS 2) respectively. The relative true positive ratios of NEWS 2 compared to NEWS were 0.98 (threshold ≥ 5) and 1.01 (threshold ≥ 7), respectively, whereas the relative false positive ratios were 0.97 (threshold ≥ 5) and 1.03 (threshold ≥ 7) respectively.

**Fig. 2:**
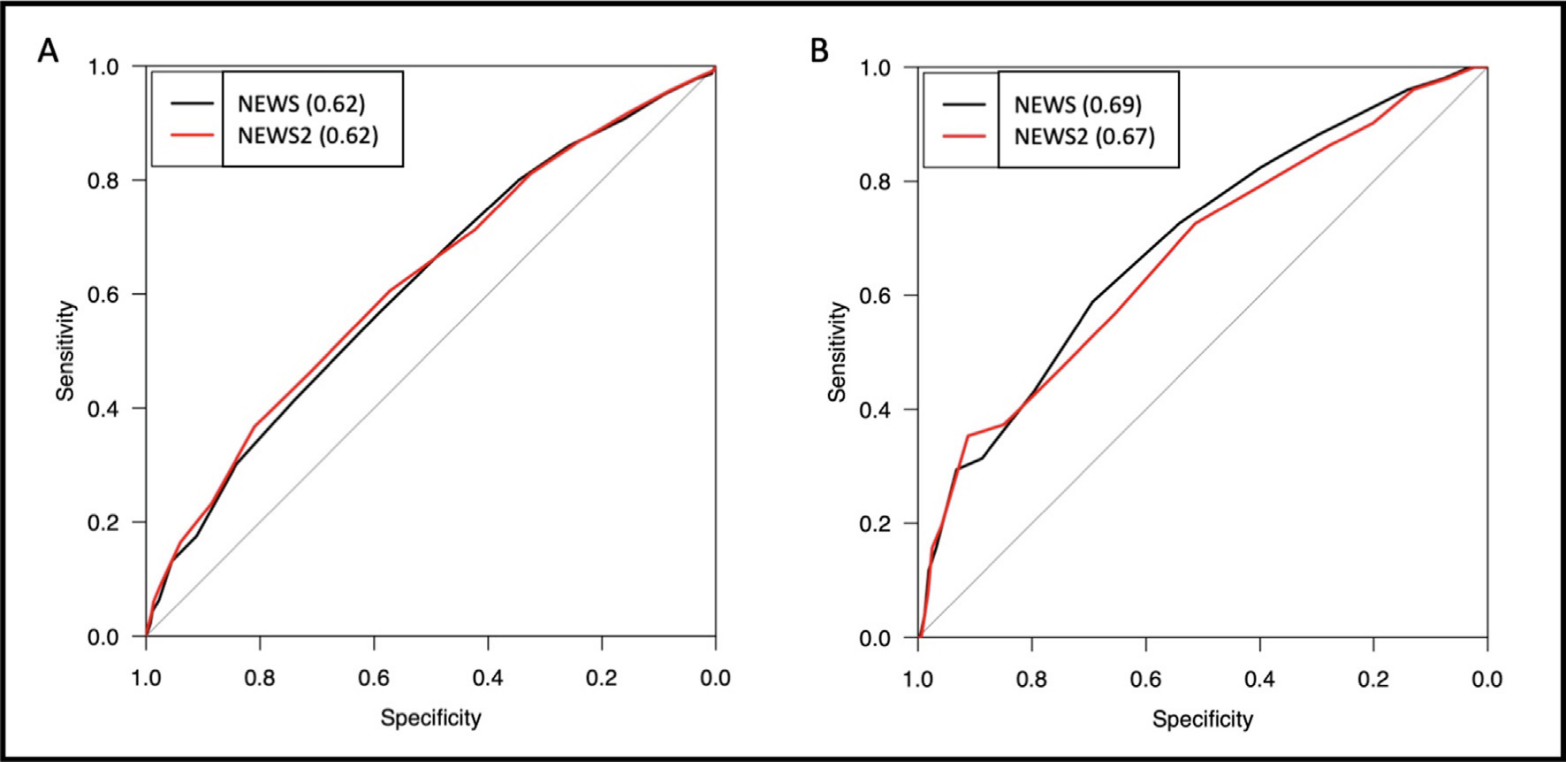
A. Area under the receiver operating characteristics (AUROC) curves for prediction of the composite outcome (unanticipated ICU admission, mortality or IHCA all within 24 hours after RRT assessment) for NEWS and NEWS 2 (AUROC 0.62/0.62) (n = 898). B. Area under the receiver operating characteristics (AUROC) curves for prediction of mortality within 24 hours after RRT assessment for NEWS (0.69) and NEWS 2(0.67) respectively (n = 898). NEWS, National Early Warning Score; RRT, Rapid response team; ICU, Intensive care unit; IHCA, in-hospital cardiac arrest.

The ability for NEWS and NEWS 2 to discriminate the composite outcome was 0.62 for both scales (Fig. 2). In discriminating mortality, the AUROC for NEWS was 0.69 and for NEWS 2 0.67 (Fig. 2). For the outcome unanticipated ICU admission, the AUROC for NEWS and NEWS 2 was 0.59 and 0.60 respectively. The AUROC for discriminating IHCA was 0.51 for NEWS and 0.47 for NEWS 2. The AUROC curves for unanticipated ICU admittance and IHCA are presented in Supplementary Fig. 1.

## Discussion

The major finding in this prospective multicentre cohort study among hospitalized deteriorating patients assessed by a RRT is the acceptable prognostic accuracy of NEWS 2 to predict mortality within 24 h. However, the predictive power to identify patients at risk of unanticipated ICU admission was rather weak and regarding IHCA the prediction was poor. Furthermore, the predictive power for the composite endpoint (i.e. unanticipated ICU admission, mortality or IHCA within 24 h) was rather weak. We found that NEWS and NEWS 2 performed similar in predicting the risk of unanticipated ICU admission, mortality or IHCA within 24 h.

The predictive power of NEWS has varied in previously published studies depending on the study settings and cohorts.^25^ As to prediction of mortality, our results are in line with Tirkkonen et al^25^ and Fernando et al^22^ who also studied RRT cohorts in contrast to the general hospitalized population; notably both studies had a longer time horizon than our study.

The ability of NEWS and NEWS 2 in this study to predict ICU admissions amongst patients assessed by the RRT was rather weak, both in absolute terms and compared to previous studies. Deranged vital signs recorded by the RRT during a review has previously been identified as an independent risk factor for 30-day mortality despite interventions of the RRT,^26^ hence a timely and adequate triage of patients in need of intensive care is of vital importance. However, decisions on ICU admittance are under the influence of many factors, e.g. competence of the RRT staff, critical care capacity and local routines and traditions when assessing need for critical care.^26^ Taking into consideration that many previous studies are retrospective, our results from a prospective study might suggest the need for a complementary, supportive triage tool in order to enable accurate decisions on the need for ICU admission during RRT assessments.

We found that both NEWS and NEWS 2 performed poorly in predicting cardiac arrest. This is in line with previous studies, showing that the discriminative ability of all early warning scores (including NEWS) are weaker in predicting cardiac arrest compared to other SAEs.^13^ One explanatory model is that the cardiac arrests are harder to predict than other SAEs due to a component of sudden cardiac arrythmias or coronary occlusions without prior deviating vital signs in IHCA.

NEWS and NEWS 2 were similar in predicting the composite outcome (e.g. unanticipated ICU-admission, IHCA and mortality within 24 h of RRT assessment). This extends results by Piementel et al,^19^ who compared the ability of NEWS and NEWS 2 to identify hospitalized patients in general wards at risk of SAEs. Those authors found no benefit of NEWS 2 in any diagnostic group compared to NEWS.^19^ However, due to their retrospective study design the documentation of the parameter “new confusion” added in NEWS 2 could not be taken into account.

The study was conducted in a cohort of RRT patients, while NEWS was originally aimed to enable early identification of patients deteriorating on a general ward. The pre-selection of patients in this study, who have already been found eligible for RRT alerting, clearly alters the conditions and most likely introduces a limitation to the validated NEWS concept.

NEWS has previously shown good to excellent discriminative ability in identifying patients in generalized hospital wards at risk of SAEs.^13^ However, in our study on patients receiving RRT assessment, NEWS and NEWS 2 performed less well. Our findings are in line with previous findings by Shappell et al,^27^ notably the outcome measure in that study was in-hospital mortality. On the other hand, Tirkkonen et al found that NEWS had a moderate predictive power as to hospital outcome in a cohort of patients attended by a RRT.^25^

There were very few patients scoring low in NEWS/NEWS 2 in the study cohort, which most likely have had an impact on the predictive power of NEWS and NEWS 2. Furthermore, the adjacent RRT assessment in our study most likely also inflicted the predictive power of NEWS and NEWS 2, since published studies implicate that the majority of RRT assessments results in treatment or intervention.^28^ Neverthless, given the limited knowledge on how to safely and effectively triage patients being assessed by RRTs, our findings add valuable insights on this group of high-risk patients.

The trade-off between the sensitivity and the specificity of an early warning score used for activation of the RRT is a delicate matter as a poor sensitivity risks to miss deteriorating patients whilst a poor specificity risks generating frequent activations resulting in alarm fatigue. This is of importance in relation to resource allocation and staff workload, considering the large number of RRT assessments performed in clinical practice.

We found a marginally higher sensitivity of NEWS 2 compared to NEWS (threshold ≥ 7), as expected at the expense of a lower specificity. Using a threshold of ≥5, the sensitivity of NEWS 2 was marginally lower compared to NEWS with a slightly higher specificity. However, a relative false positive ratio of 1.03 (NEWS 2/NEWS, threshold ≥ 7) and 0.97 (NEWS 2/NEWS, threshold ≥ 5) respectively may suggest a similar risk of “false alarms” when comparing NEWS and NEWS 2.

Future research should focus on improving the identification of high-risk patients among those being assessed by RRTs without increasing the workload for the medical staff. In pursuit of a decision support in the form of a new prediction model one may consider adding biochemical markers, age, comorbidity and information extracted from electronical health records. Also, the rapid development of artificial intelligence will likely facilitate real-time analyses when creating accurate and simple future prediction models for risk assessment.

### Strengths and limitations

This is a large, prospective cohort study, covering 26 hospitals in Sweden, with a rigorous study protocol and a low rate of missing variables. The uniform calculation of both NEWS and NEWS 2 scores by the study team constitutes another strength. However, there are several limitations. We did not retrieve any information about comorbidities, functional status, or frailty. There may also have been variations across hospitals with regards to measurements of vital parameters. Furthermore, some participating hospitals have High dependency units, whereas patients in other hospitals would have been admitted directly to the ICU. This may have contributed to a slight underestimation of the number of unanticipated ICU admissions.

## Conclusion

The prognostic accuracy of NEWS 2 to predict mortality within 24 h was acceptable in patients being assessed by a RRT. The predictive power to identify patients at risk of unanticipated ICU admission or the composite endpoint was rather weak, and the predictive power to identify patients at risk for IHCA was poor.

Whereas NEWS and NEWS 2 performed similar in predicting the risk of SEAs, their performances were not sufficient to enable use as a risk stratification tool among patients being assessed by a RRT.
