# Supplementary material for: The predictive power of the National Early Warning Score (NEWS) 2, as compared to NEWS, among patients assessed by a Rapid response team: A prospective multi-centre trial
Source: Resusc Plus. 2021 Dec 24;9:100191. doi: 10.1016/j.resplu.2021.100191 (PMC8718668; doi:10.1016/j.resplu.2021.100191)
Supplement: Supplementary data 1 [file mmc1.docx]

## Supplementary information

**Additional file 1. Table S1:** Participating hospitals (n=26); demographics, RRT availability and number of RRT assessment during the study period.

**Additional file 2. Table S2:** Symptom/diagnosis upon admission in detail (n=898).

**Additional file 3. Table S3:** Primary reason for RRT assessment in detail (n=898).

## Supplementary data

| Table S1  Participating hospitals (n=26); demographics, type of EWS, RRT availability, number of RRT assessments and number of included patients during the study period. | | | | | | | | |
| --- | --- | --- | --- | --- | --- | --- | --- | --- |
|  | Hospital category | EWS | # Beds | # ICU Beds | HDU (Y/N) | RRT 24/7 (Y/N) | # RRT | # Included patients |
|  | **Total** |  | **8181** | **215** |  |  | **1279** | **965** |
| 1 | University | NEWS 2 | 571 | 16 | Yes | Yes | 119 | 106 |
| 2 | University | NEWS | 560 | 8 | Yes | No | 125 | 30 |
| 3 | University | NEWS 2 | 526 | 20 | Yes | Yes | 160 | 97 |
| 4 | University | NEWS 2 | 500 | 8 | Yes | Yes | 26 | 24 |
| 5 | University | NEWS 2 | 490 | 21 | Yes | Yes | 48 | 43 |
| 6 | University | NEWS | 473 | 9 | Yes | Yes | 53 | 35 |
| 7 | University | NEWS | 470 | 10 | Yes | Yes | 115 | 96 |
| 8 | University | NEWS | 439 | 10 | No | Yes | 21 | 15 |
| 9 | University | NEWS, ** | 330 | 8 | Yes | Yes | 29 | 22 |
| 10 | University | NEWS 2 | 294 | 5 | Yes | Yes | 45 | 38 |
| 11 | County | NEWS | 453 | 12 | Yes | Yes | 148 | 102 |
| 12 | County | NEWS 2 | 339 | 8 | Yes | Yes | 34 | 30 |
| 13 | County | NEWS 2 | 300 | 6 | No | Yes | 17 | 16 |
| 14 | County | NEWS 2 | 273 | 7 | No | Yes | 34 | 32 |
| 15 | County | NEWS | 250 | 6 | No | Yes | 42 | 41 |
| 16 | County | NEWS | 240 | 7 | No | Yes | 41 | 30 |
| 17 | County | NEWS 2 | 220 | 6 | No | Yes | 38 | 35 |
| 18 | County | NEWS 2 | 219 | 6 | No | Yes | 37 | 36 |
| 19 | County | NEWS 2 | 215 | 11 | No | Yes | 35 | 31 |
| 20 | County | NEWS 2 | 200 | 6 | No | Yes | 31 | 27 |
| 21 | County | NEWS 2 | 184 | 6 | Yes | Yes | 36 | 35 |
| 22 | District | NEWS, * | 317 | 6 | No | Yes | 9 | 8 |
| 23 | District | NEWS 2 | 110 | 4 | No | Yes | 21 | 20 |
| 24 | District | NEWS 2 | 107 | 3 | Yes | Yes | 4 | 4 |
| 25 | District | NEWS 2 | 60 | 3 | No | Yes | 6 | 6 |
| 26 | District | NEWS 2 | 41 | 3 | No | Yes | 5 | 6 |

| * NEWS 2 from January 1, 2020 |
| --- |
| ** NEWS 2 from January 21, 2020 |

EWS, early warning score; NEWS, National Early Warning Score; ICU, intensive care unit; RRT, rapid response team; HDU, high dependency unit.

| **Table S2**  Symptom/diagnosis at admission (detail). Data are presented as numbers (percentages and cumulative sum). | | | |
| --- | --- | --- | --- |
| Variable | Value (n=898) |  |  |
| Surgical diseases | 139 | 15.5 | 15.5 |
| Other infections | 89 | 9.9 | 25.4 |
| Orthopedic diseases | 76 | 8.5 | 33.9 |
| Pneumonia and respiratory infections | 74 | 8.2 | 42.1 |
| Sepsis | 73 | 8.1 | 50.2 |
| Other cause of admission | 56 | 6.2 | 56.5 |
| Dyspnoe | 46 | 5.1 | 61.6 |
| Abdominal pain | 42 | 4.7 | 66.3 |
| Malignancy | 28 | 3.1 | 69.4 |
| Cardiovascular diseases | 25 | 2.8 | 72.2 |
| Respiratory diseases | 24 | 2.7 | 74.8 |
| Altered level of consciousness | 22 | 2.4 | 77.3 |
| Catastrophic conditions | 21 | 2.3 | 79.6 |
| Gastrointestinal bleeding | 18 | 2.0 | 81.6 |
| Neurological diseases | 17 | 1.9 | 83.5 |
| Impaired general condition | 15 | 1.7 | 85.2 |
| Intoxication | 14 | 1.6 | 86.7 |
| Gastrointestinal diseases | 13 | 1.4 | 88.2 |
| Malignancy and infection in combination | 11 | 1.2 | 89.4 |
| Chest pain | 10 | 1.1 | 90.5 |
| Trauma | 10 | 1.1 | 91.6 |
| Respiratory diseases and infection | 10 | 1.1 | 92.8 |
| Psychiatric diseases | 8 | 0.9 | 93.7 |
| Haematological diseases | 8 | 0.9 | 94.5 |
| Renal diseases | 7 | 0.8 | 95.3 |
| Diabetic emergencies | 6 | 0.7 | 96.0 |
| Metabolic or endocrine diseases. electrolytes | 6 | 0.7 | 96.7 |
| Ascites | 6 | 0.7 | 97.3 |
| Respiratory and cardiovascular diseases in combination | 4 | 0.4 | 97.8 |
| Tromboembolic diseases | 3 | 0.3 | 98.1 |
| Ophtalmological diseases | 3 | 0.3 | 98.4 |
| Syncope | 2 | 0.2 | 98.7 |
| Rheumatological diseases | 2 | 0.2 | 98.9 |
| Gynecological diseases | 2 | 0.2 | 99.1 |
| Swollen legs | 1 | 0.1 | 99.2 |
| Missing data | 7 | 0.8 | 100.0 |

Catastrophic conditions include aortic aneurysm (ruptured), aortic dissection, circulatory failure, cardiac arrest, cardiogenic shock, hypertensive crisis, hypothermia, intracerebral haemorrhage, multiple organ failure and suicide (hanging).

| **Table S3**  Other cause of RRT assessment (detail, n=139). Data are presented as numbers (percentages). | |
| --- | --- |
|  |  |
| Respiratory distress | 42 (30) |
| Circulatory distress | 37 (27) |
| RRT follow- up | 15 (11) |
| Mental status change | 10 (7.2) |
| Blood test result abnormality | 7 (5) |
| Altered diuresis | 5 (3.6) |
| Assessment need for intensive care | 4 (2.9) |
| Assessment need of dialysis | 2 (1.4) |
| Sepsis/ suspected sepsis | 2 (1.4) |
| Pain, no further specification | 2 (1.4) |
| Gastrointestinal bleeding | 2 (1.4) |
| Chestpain | 1 (0.7) |
| Bleeding, no further specification | 1 (0.7) |
| Bleeding from urinary catheter | 1 (0.7) |
| Epileptic seizure | 1 (0.7) |
| Preoperative assessment | 1 (0.7) |
| Arterial Catheterization | 1 (0.7) |
| Discussion level of care | 1 (0.7) |
| Abdominal pain | 1 (0.7) |
| Severe abdominal and back pain | 1 (0.7) |
| Epileptic seizure and desaturation | 1 (0.7) |
| High need for insulin, cPAP need, fatigue | 1 (0.7) |

RRT, rapid response team.


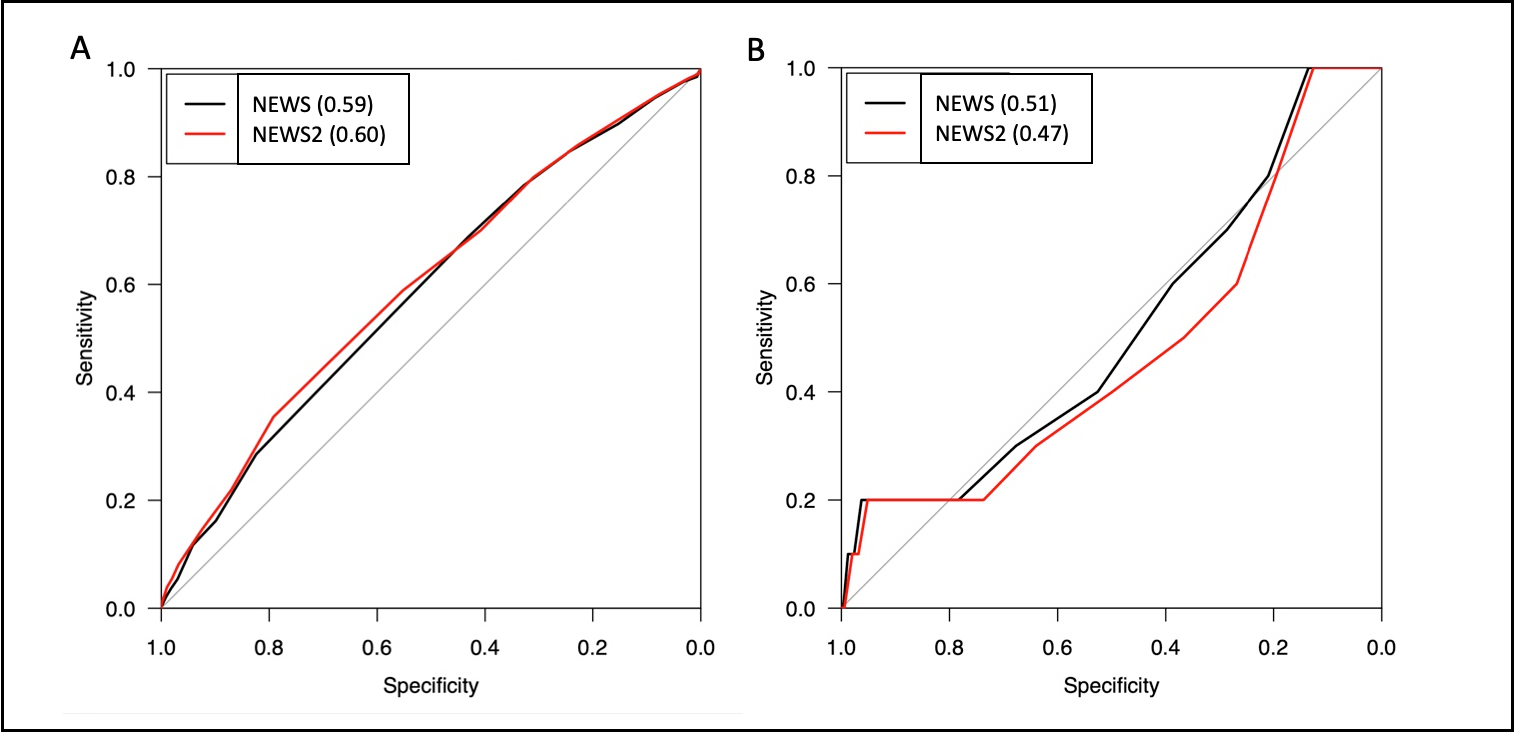


Figure S2A: Areas under the receiver operating characteristics (AUROC) curves for prediction of unanticipated ICU admission within 24 hours after RRT assessment for NEWS and NEWS 2 (AUROC 0.59 and 0.60 respectively) (n=898).

Figure S2B: Area under the receiver operating characteristics (AUROC) curves for prediction of IHCA within 24 hours after RRT assessment for NEWS and NEWS 2 (AUROC 0.51 and 0.47 respectively) (n=898).

NEWS, National Early Warning Score; RRT, rapid response team; ICU, intensive care unit; IHCA, in-hospital cardiac arrest.
